# Autoimmune pancreatitis—New evidence for clinical management strategies

**DOI:** 10.1002/ueg2.12537

**Published:** 2024-01-25

**Authors:** C. Ammer‐Herrmenau, J. Hamm, A. Neesse

**Affiliations:** ^1^ Department of Gastroenterology, Gastrointestinal Oncology and Endocrinology University Medical Center Goettingen Goettingen Germany

**Keywords:** autoimmune pancreatitis, chronic inflammatory bowel disease, pancreatic cancer risk, SARS‐Cov‐2, vaccination

## INTRODUCTION

Autoimmune pancreatitis (AIP) is a rare inflammatory condition of the pancreas with an incidence of one case per 100,000 inhabitants in Europe.^1^ According to International Consensus Diagnostic Criteria, AIP is categorized into type 1, type 2, and not otherwise specified (NOS).^2^ While type 1 (male predominance) is considered as systemic IgG4 disease with common involvement of the bile duct and other organs such as the kidneys and the salivary glands, type 2 occurs in younger adults (30–40 years) without any gender preference. Recently, type 3 was introduced as a side effect of checkpoint inhibitors.^3^ One clinical hallmark of all AIP types is the responsiveness to corticosteroids. However, type 1 is accompanied with a higher rate of relapse and steroid dependence. As AIP is considered a rare disease, and accurate diagnosis and subsequent clinical management is often challenging and may result in unnecessary pancreatic resections, premature steroid cessation or missed diagnosis of pancreatic ductal adenocarcinoma (PDAC). Two recently published articles in the United European Gastroenterology Journal provide compelling insights into key aspects of clinical management strategies of AIP patients that broaden our understanding of this complex and rare disease entity.^4^, ^5^

## LONG TERM FOLLOW‐UP DATA FROM PATIENTS WITH AIP TYPE 2

De Pretis et al. present long‐term follow‐up data from 88 patients with AIP type 2.^4^ Notably, this is the largest reported AIP type 2 cohort to date with a mean follow‐up of 9.2 ± 7.1 years. Intriguingly, the authors found a higher proportion of male patients (62.5%) affected by AIP type 2 contradicting the paradigm that there is no gender preference.^6^, ^7^, ^8^ The most common clinical presentation at diagnosis was an episode of acute pancreatitis and weight loss (47% and 49% respectively). Notably, six patients were asymptomatic at the time point of diagnosis and were only detected as incidental findings during follow‐up examinations of ulcerative colitis (UC). Interestingly, almost 90% of AIP type 2 cases were associated with UC (87.5%). In 2/3 of all cases, UC was either diagnosed simultaneously or preceded the diagnosis of AIP with an average of 5 years. However, 1/3 of the patients developed UC after AIP onset with an average delay of more than 3 years, a finding that could only be made performing such a meticulous long‐term follow‐up study. This is important for clinical management strategies as patients with idiopathic pancreatitis or NOS and no history or clinical signs of inflammatory bowel disease (IBD) should be considered for faecal calprotectin and/or screening endoscopy to detect clinically silent gastrointestinal inflammation.

Besides the association with IBD that facilitates the definite diagnosis of AIP type 2, histological confirmation proved to be challenging (diagnostic accuracy of endoscopic ultrasound [EUS] only 31%) even in a large volume pancreas centre such as the University of Verona pointing to the necessity to improve EUS‐guided fine needle biopsy (EUS‐FNB) approaches to further reduce the high rate of unnecessary surgical resections. This is particularly important as most patients responded well to steroids and the relapse rate was low. Endocrine and exocrine insufficiency (around 10%) was lower as compared to other recently published studies,^9^ but increased in surgically resected patients. Notably, none of the patients developed PDAC during the observation period which is in line with existing literature,^9^ providing evidence that a life‐long pancreas specific follow‐up may not be necessary in all AIP type‐2 patients. Furthermore, overall relapse rate was low (13%) and did not increase beyond 5 years.

We believe that clinicians should draw the following conclusions from this important long‐term outcome study:

1. Screen for IBD in patients with idiopathic pancreatitis as AIP max precede IBD in up to 30% of cases.
2. Improve diagnostic accuracy of EUS‐FNB for suspected AIP to avoid unnecessary surgery.
3. Consider termination of a pancreas‐specific follow‐up surveillance beyond 5 years due to negligible relapse and PDAC risk.

## SARS‐COV‐2 VACCINATION IS SAFE IN AIP 2 PATIENTS

The increased risk of immunocompromised patients to suffer from severe SARS‐Cov‐2 infections is well known. Furthermore, SARS‐Cov‐2 infections as well as SARS‐Cov‐2 vaccinations have been associated with the exacerbation or new onset of autoimmune disease such as diabetes, colitis or pancreatitis. Systematic observations of mRNA‐based vaccinations are currently lacking in AIP patients. In this issue, Perkhofer et al., report on the safety and effectiveness of SARS‐Cov‐2 vaccinations in 30 patients with AIP (type 1 *n* = 16, type 2 *n* = 14).^5^ Most patients received at least two and 43% three or even 4 vaccinations. The reported side effects were highly similar to the age‐matched healthy control group (*n* = 159). 2/3 of all patients did not have any vaccine‐related side effects, 13% had minor local and 20% suffered from self‐limiting systemic symptoms. Notably, AIP‐related symptoms did not aggravate after vaccination. Seventeen patients reported a confirmed SARS‐Cov‐2 infection, however, only one patient was admitted to the hospital due to pneumonia. Overall, the risk of getting infected with SARS‐Cov‐2 and the disease severity was comparable to the healthy population. These findings highlight the safety and effectiveness of the vaccination and provide an evidence‐based approach for AIP patients.

## CONFLICT OF INTEREST STATEMENT

The authors have no conflicts of interest to declare.

## Data Availability

Data sharing is not applicable to this article as no new data were created or analyzed in this study.
